# Differential expression of progesterone receptor isoforms related to *** PGR*** +331g/a polymorphism in endometriosis: A case-control study

**DOI:** 10.18502/ijrm.v17i3.4517

**Published:** 2019-05-29

**Authors:** Sepideh Mousazadeh, Azadeh Ghaheri, Maryam Shahhoseini, Reza Aflatoonian, Parvaneh Afsharian

**Affiliations:** ^1^ Department of Tissue Engineering and Regenerative Medicine, Faculty of Advanced Technologies in Medicine, Iran University of Medical Sciences, Tehran, Iran.; ^2^ Department of Genetics, Reproductive Biomedicine Research Center, Royan Institute for Reproductive Biomedicine, ACECR, Tehran, Iran.; ^3^ Department of Epidemiology and Reproductive Health, Reproductive Epidemiology Research Center, Royan Institute for Reproductive Biomedicine, ACECR, Tehran, Iran.; ^4^ Department of Endocrinology and Female Infertility, Reproductive Biomedicine Research Center, Royan Institute for Reproductive Biomedicine, ACECR, Tehran, Iran.

**Keywords:** *Endometriosis*, * Progesterone receptor A*, * Progesterone receptor B*, * rs10895068.*

## Abstract

**Background:**

Endometriosis are defined as a progesterone-resistance disease. Two progesterone receptor (PR) isoforms, namely PR-A and PR-B, mediate the special effects of progesterone. One of the most effective polymorphism in the promoter region of *PGR* is the +331G/A.

**Objective:**

The differential expression level of PR isoforms due to +331G/A polymorphism may be able to influence the function of progesterone and reduce the susceptibility of endometriosis.

**Materials and Methods:**

This analytic, case-control study was carried out at Royan Institute, Tehran, Iran. Whole-blood samples were collected from 98 infertile women undergoing laparoscopy for endometriosis and 102 healthy fertile women. After DNA extraction, genotype frequencies were determined by polymerase chain reaction-restriction fragment length polymorphism. Then, RNA was extracted from the selected eutopic tissue samples of endometriosis patients. Analysis of PR-A and PR-B mRNA expressions were performed using Real-time polymerase chain reaction.

**Results:**

The frequency distribution of GG, GA genotypes in +331G/A polymorphism was 98.04%, 1.96% in the patients and 97.96%, 2.04% in the control groups, respectively (p = 0.968). Although our data did not show any significant association with +331G/A in the patient and control groups, we were able to demonstrate significantly higher expression level of PR-B and no significant lower expression level of PR-A isoforms in patients by favoring GA to GG genotypes (p = 0.017, p = 0.731, respectively).

**Conclusion:**

Our findings show that patients with GA genotypes had a higher expression level of PR-B compared to patients with GG genotypes.

## 1. Introduction

Infertility reduces the quality of life for millions of women worldwide, causing significant annual costs in the health care system (1, 2). To our knowledge, endometriosis involves 30–50% of women with infertility (3). Endometriosis disease is defined as a benign gynecological disorder, which is characterized by the being of endometriotic lesions containing of functional endometrial glands and stroma outside of the uterine cavity (4, 5). Endometriosis affects natural fertility through various approaches, including physical blocking of fallopian tubes, endocrines, and oocyte deficiency, like luteinized unruptured follicle syndrome, impaired folliculogenesis, luteal phase defect, and premature or multiple luteinizing hormone surges (6, 7). However, the association of endometriosis and infertility has been discussed yet and is still unclear (6). The high level of activate macrophages, prostaglandins, interleukin 1 (IL-1), tumor necrosis factor (TNF) and proteases in the endometriosis may have opposed effects on the ovulatory, sperm, embryo, or fallopian tube, causing infertility (6, 7). A further potential mechanism for these `inflammation activities' mediates by an interaction between the pro-inflammatory nuclear factor-κB (NF-κB) transcription factor complex and sex steroid progesterone hormone in human myometrial cells (8). In fact, endometriosis are known as a progesterone resistance disease (9). The genomic effects of progesterone upon target tissues are normally mediated by nuclear Progesterone Receptor (PR) isoforms specifically PR-A and PR-B (10). These isoforms are encoded from a single gene by differential promoter usage on chromosome 11q22-q23 (10, 11). PR-A is a truncated (by 164 N terminal amino acids) form of PR-B isoform (10). The capacity of these isoforms to evaluate the transcriptional activity of progesterone plays a major role in the biology of endometriosis (8, 9). Whereas PR-B isoform was shown to stimulate transcriptional activity, PR-A isoform seemed to act as a dominant repressor of PR-B in many target tissues (9). Consequently, altered PR-A/PR-B ratio might render responsive or resistant to progesterone in specific target tissues, which could be essential in pathogenesis and inflammation activity of endometriosis.

Recently, A putative functional polymorphism in the promoter region of the *PGR* gene has been identified in the position +331 (G+331A) (rs10895068) (11, 12), creating another TATA box, which provides a specific transcriptional start site by favoring high transcriptional production of PR-B compared to PR-A isoform (11, 12). PR-B deficiency isoform may also be main reason for other consequences of progesterone resistance in endometriosis (13, 14).

Our objective was to determine how the PR-A/PR-B ratio alters via +331G/A polymorphism in endometriosis because this alternation can affect the capacity of progesterone response to modulate the expression of inflammatory mediators and influence endometriosis disease.

## 2. Materials and Methods

### Study populations

A total of 200 women, 98 infertile women undergoing endometriosis were recruited from July 2012 to March 2015 at Roran Institute, Iran. All of these women with endometriosis confirmed by laparoscopy and postoperative histology study were included as the patient group. Our control subjects were 102 healthy, fertile women (without endometriosis) with at least one child, having regular cycles, and no evidence of any pathologic uterine disorder. None of them had visible endometrial hyperplasia or neoplasia, inﬂammatory or autoimmune disease, and endometriosis at the time of clinical examination. None of the patients and control subjects had received hormonal treatments for at least three months prior to surgery. The distribution of the samples according to the mean age and Body Mass Index was compared with women with/without endometriosis (control group) that showed no statistical differences (Table I).

### Polymorphism study

#### Polymerase chain reaction condition

Whole-blood samples were collected from all 200 subject women. Genomic DNA was extracted from whole peripheral EDTA-blood samples by using the standard salting-out method from patient and control samples (15). The DNA was stored at 4${}^{\circ }$C until analyzed. Polymerase chain reaction-restriction fragment length polymorphism (PCR-RFLP) was used to genotype the +331G/A promoter SNP (Single Nucleotide Polymorphism). The PCR primers were designed to amplify the specific PR gene fragment. The expected product-size was shown in Table II. The PCRs were performed in a total volume of 30 μl containing 50 ng of genomic DNA, 4 pmol of each primer, and 7.5 μl master mix (Amplicon Taq DNA Polymerase 2x master mix (red), 1.5 mM MgCl${}_{2}$). PCR protocol consisted of three steps: primary denaturation at 94${}^{\circ }$C for 4 min continued by 33 cycles at 94${}^{\circ }$C for the 30 s, annealing at 67${}^{\circ }$C for 30 sec, extension at 72${}^{\circ }$C for 1 min, and a final extension at 72${}^{\circ }$C for 10 min. After amplification, the products of PCRs were assessed by 1.2% ultra-pure agarose gel electrophoresis and visualized by ethidium bromide staining followed by Molecular ImagerⓇ Gel Doc${}^{TM}$ XR+.

#### RFLP

**Figure 1:**
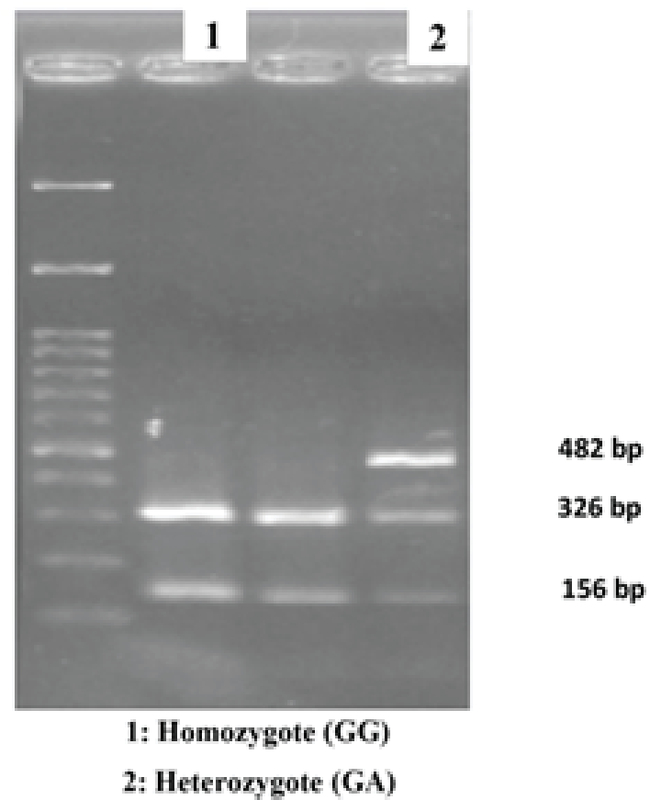
The RFLP pattern of *PGR* promoter +331G/A polymorphism showing two types of homozygotes (GG) and a heterozygote (GA)

Identification of +331G/A promoter polymorphism was based on RFLP technique of fragments that had been amplified by PCR. The amplified PCR fragment was digested with *N1aIV *restriction endonuclease (Fermentase Biolabs, MA, USA) overnight at 37${}^{\circ }$C. The size of the digestion products is evaluated by 1.7% ultra-pure agarose gel electrophoresis that was explained earlier (Figure 1); 10% of samples of different genotypes were randomly sequenced by a DNA sequencing Sanger method (Sequetech Inc., USA) to confirm genotyping results. Sequenced results were blasted against the ancestral sequence in NCBI (http://blast.ncbi.nlm.nih.gov.com) and diagrams were analyzed via Finch TV software version 1.4.0.

### Expression assay

Out of 98 infertile women with endometriosis, eutopic (endometrium) tissue samples were recruited from eight women, including two patients with GA (one of them was in proliferative phase and another one was in secretory phase of the cycle) and six patients with GG genotypes. According phases of the cycle in patients with GA genotypes, three of tissue samples were recruited in proliferative phase and another half were in secretory phase of the cycle in patients with GG genotypes. Tissue samples were obtained by pipelle from all same patients.

### RNA extraction and cDNA preparation

In brief, RNA was isolated from frozen tissue samples using Trizol (Invitrogen, USA) following the manufacturer's recommendations. One microgram of genomic DNA for each sample was treated with RNase free DNase I (#EN0521-Fermentas, Thermo scientific, Germany), incubation at 37${}^{\circ }$C for 30 min. DNase I enzyme was inactivated by EDTA (50 mM, Fermentas, Thermo Scientific, Germany) and incubation at 65${}^{\circ }$C for 7 min. Complementary DNAs (cDNAs) were prepared from one microgram of total RNA for each sample by One-step RT-PCR, using first-strand cDNA synthesis kit (K1632- Fermentas, Thermo Scientific, Germany). Synthesized cDNA was stored at -20${}^{\circ }$C until used.

### Quantitative real-time PCR

Analysis of PR-A and PR-B mRNA expressions were performed using SYBRⓇ Premix Ex Taq II (Applied Biosystems, USA) on a Light Cycler System, 7500 software version 2.0.1 (Applied Biosystems, 7500, USA) as recommended by the manufacturer. The specific primers used for amplification of PR-A, PR-B, and β-actin (as a housekeeping gene) were designed using specific primer analysis software the Gene Runner (version 3.05) and Perl Primer software (version v1.1.20). These sequences were analyzed by Nucleotide Blast and Primer-Blast in the NCBI database (http://blast.ncbi.nlm.nih.gov/). The primers used in this study and the expected product-sizes from them were shown in Table II. Primers were purchased by order from Pishgam Co, Iran.

Each assay was carried out in a final volume of 20 µl containing 10 µl SYBRⓇ Premix Ex Taq II (consisting of Taq DNA polymerase reaction buffer, dNTP mix, SYBR Green II, MgCl${}_{2}$, and Taq DNA polymerase), 5 pmol (Pico mole) of β-actin, PR-A or PR-B primers, 25 ng/µl of synthesized cDNA and water to reach. Each sample was analyzed in duplicate in three independent real-time RT–PCR assays. The amplification program consisted of the following three steps. Cycling conditions included incubation at 95${}^{\circ }$C for 10 min, and 40 cycles of 95${}^{\circ }$C for 15 sec (denaturation), 60${}^{\circ }$C for 1 min (annealing) for β-actin, PR-A, and PR-B. Finally, the temperature was raised gradually from 60${}^{\circ }$C to 95${}^{\circ }$C for 15 sec and 1 min at 60${}^{\circ }$C for melting curve analysis. After each run, a melting curve analysis was achieved to verify the specificity of the PCR reaction. All samples were retested with a cycle threshold coefficient of variation value higher than one degree. To confirm the melting curve results, we assayed representative samples of the real-time PCR products on 2% ultra-pure agarose (Invitrogen, USA) gel electrophoresis (Paya Pazhoh Pars, Iran), and stained them with ethidium bromide (Sigma Aldrich, USA) prior to visualization on a Molecular ImagerⓇ Gel Doc${}^{TM}$ XR+ (BioRad, USA).

**Table 1:** Clinical characteristics of groups.


**Groups**	**Number**	**Age (yr)**	**BMI (kg/m${}^{2}$)**	**Stage (n)**	**During of Infertility**
Controls	102	29 ± 11	27.6 ± 8.1	- -
Patients	98	31 ± 10	24.5 ± 6.1	I (6), II (12)III (34),IV (46)	> 24 months
P-value	- p > 0.05	p > 0.05	–	–
Note: Data are expressed as mean ± SEM and values in parentheses are percentages; BMI: Body mass index					

**Table 2:** Primer sequences of +331G/A SNP, PR-A, PR-B and β-actin.


**Name**	**Primer**	**PCR Product (bp)**
SNP (PGR)	(Forward)5’-GCAGTACGGAGCCAGCAGAAGT-3’	
	(Reverse)5’-AGAGGGAGGAGAAAGTGGGTGTTGA-3’	<brow>-2</erow> 492
PR-A	(Forward) 5’-AATGGAAGGGCAGCACAACT-3’	
	(Reverse) 5’-TGTGGGAGAGCAACAGCATC-3’	<brow>-2</erow>192
PR-B	(Forward) 5’-AAGGGGAGTCCAGTCGTCAT-3’	
	(Reverse) 5’-CGAAACTTCAGGCAAGGTGT-3’	<brow>-2</erow>165
β-actin	(Forward) 5'-CAAGATCATTGCTCCTCCTG-3'	
	(Reverse) 5'-ATCCACATCTGCTGGAAGG-3'	<brow>-2</erow>90

### Ethical consideration

This population-based study was approved by the Department of Genetics in Royan Institute, Tehran, Iran. Approval was obtained from the Institutional Research Ethics Board. The Ethics Committee of Royan Institute approved this study (No: EC/93/1047). All participants provided a signed informed consent.

### Statistical analysis 

*PGR* genotypes and population's clinical information were compared between groups (endometriosis and control) using independent *t*-test and chi-square test. The expression levels of PR-A and PR-B in patients bearing GA or GG genotypes were compared using Wilcoxon in extracting eutopic samples. Statistical analysis was conducted using SPSS software (Statistical Package for the Social Sciences, version 16.0, SPSS Inc., Chicago, IL, USA). All statistical tests were two-tailed and a p < 0.05 was considered statistically significant.

## 3. Results

### Polymorphism of *** PGR*** promoter

The frequency of distribution of GG and GA genotypes in +331G/A polymorphism in 200 blood samples of patients was 98.04% and 1.96%, and in the control group, it was 97.96%, 2.04%, which was not significantly different (p = 0.968).

Eight of ninety-eight patients underwent tissue sampling for expression study, the results of a study of their polymorphism showed two and six patients with GA and GG genotypes, respectively.

#### The expression of PR isoforms via +331G/A promoter polymorphism

**Figure 2:**
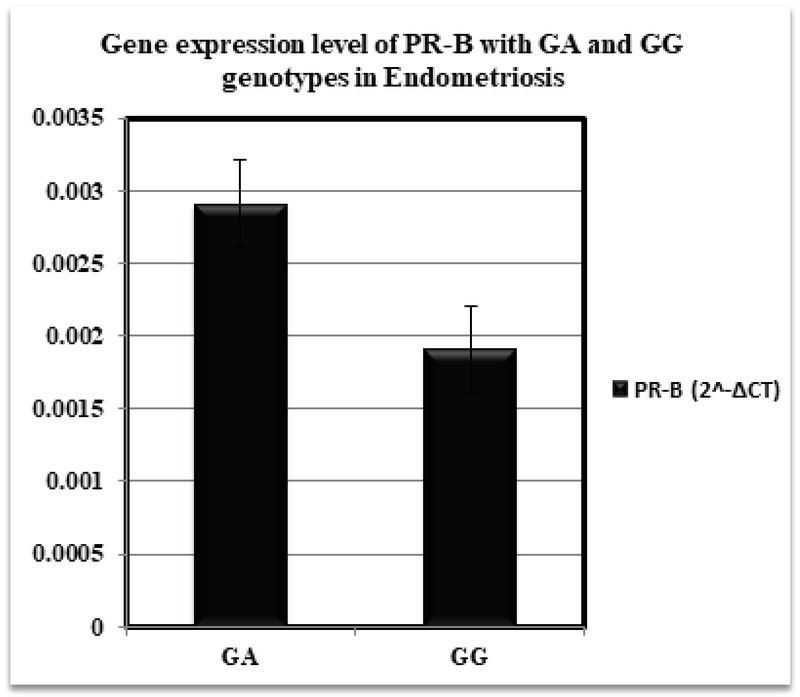
The expression level of PR-B isoform via +331G/A promoter polymorphism in endometriosis

**Figure 3:**
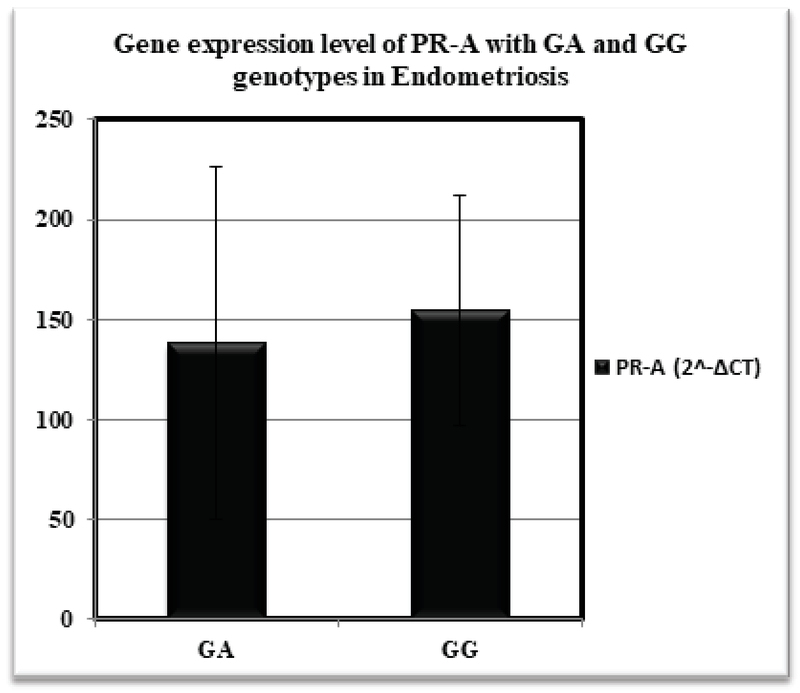
The expression level of PR-A isoform via +331G/A promoter polymorphism in endometriosis

Our results demonstrated significantly higher expression level of PR-B isoform in the endometrium of patients bearing GA compared to subjects with GG genotypes in +331G/A polymorphism of *PGR* gene (0.0029 ± 0.0003 vs. 0.0019 ± 0.0003; p = 0.017) (Figure 2). The expression level of PR-A in eutopic endometrium of patients was not significantly different between GA and GG genotypes (138.27 ± 87.84 vs. 155.01 ± 57.50; p = 0.731) (Figure 3).

## 4. Discussion

### The effects of progesterone receptors in endometriosis

Progesterone acts by binding to and activating PR-A and PR-B isoforms expressed in target tissues (16). These isoforms are functionally different and encoded from a single gene by differential estrogen-regulated promoters (17). PRs are ligand-induced transcription factors (16, 18). These isoforms contain of DNA-binding domain (DBD), inserted among an upstream N-terminal region that consists the activation (AF) and inhibitory (IF) roles, a downstream hinge region and C-terminal ligand-binding domain (LBD) (19). Just only deference of PR-A and PR-B isoforms is that PR-B isoform contain an extra 164 amino acid far N-terminal region, called the “B-upstream segment” (BUS) by advising AF3 activity (16). This part is missing in PR-A isoform. Thus, the global structural differences between PR-B and PR-A are distended from the BUS that controls their properties (16). Several recent studies are indicative of resistance to progesterone action in endometriosis (14). The effects of progesterone upon target tissues are generally mediated through PR-A/PR-B ratio to modulate the expression of genes, especially the inflammation-associated transcription factor NF-κB (8, 10). The molecular mechanism of progesterone resistance in endometriosis may be related to reduction, or lack, of the PRs, especially PR-B isoforms (9, 14). In normal endometrium, progesterone induces the expression of the enzyme 17β-hydroxysteroid dehydrogenase type 2 (17β-HSD-2), which metabolizes the biologically active estrogen estradiol (E2) to estrone (E1) (20). Progesterone does not induce epithelial 17β-HSD-2 expression may be due to the lack of PR-B in the endometriotic lesion (14, 20). This defect causes the deficient metabolism of E2 and increases local concentrations of E2 in endometriosis (20). The best-defined mitogen for the growing and inflammation activity of ectopic lesions is active E2 (14). On the other hand, progesterone exerts anti-inflammatory actions by inhibiting the pro-inflammatory NF-κB transcription factor complex in human myometrial cells (8, 21). Progesterone increases expression of inhibitor-κBα, a repressor of NF-κB transcription factor, via PR-B isoform, therefore inhibits pro-inflammatory gene expression (8). The crosstalk between oocytes and sperms may is blocked during fertilization by these inflammation activities due to progesterone resistance and reduced sperm affinity to the zona pellucida through TNF, IL-1, migration inhibitory factor, oxidative stress, and the RANTES cytokine in endometriosis (6, 22). The high level of activate macrophages, prostaglandins, IL-1, TNF, and proteases in the endometriosis could have opposing effects on the evolution of ovulatory, sperm, and embryo causing infertility in endometriosis (6, 22). In the present study, we demonstrated the expression levels of PR-A and PR-B via +331G/A promoter polymorphism in endometriosis. Altered PR-A/PR-B ratio via +331G/A polymorphism may affect the capacity of progesterone response to modulate the expression of inflammatory mediators and influence the pathogenesis of the disease.

### The association between a *** PGR*** promoter polymorphism and endometriosis

Recently, A putative new functional polymorphism has been recognized in the promoter region of the PGR gene in the position +331 (G+331A) (12). The +331A allele makes a potential TATA-box, creating a specific transcriptional start site, that increases expression level of PR-B isoform (12, 23). Consequently, it could be reason of high expression level of PR-B isoform by favoring A allele. Near et al have been reported a decreased level of PR-A to PR-B expressions in the +331 A allele and if the reported association with endometriosis is confirmed, it would suggest that this ratio could be important in endometriosis (11). To our knowledge, the biochemical studies suggest that +331G/A PGR polymorphism may contribute to endometrial cancer risk through increased PR-B-dependent stimulation of endometrial cell growth (24). An alteration in the PR-A/PR-B ratio by the overexpression of PR-B isoform due to +331G/A promoter polymorphism may lead to an increased risk of developing overall female cancer risk (25). Although Berchuck and colleagues have suggested a reduced risk of endometriosis associated with A allele of +331 SNP (26), Treloar et al found no association (27). Van Kaam et al informed a low risk of endometriosis in women who had +331A allele compared to women carried +331G once (28), but this observation is in the directional conflict with reported that was studied by Gentilini and colleagues (29). Part of the aim of this study was to investigate the association between the putative functional promoter +331G/A SNP and endometriosis. Here we present that the results of our analysis suggest that +331G/A in the promoter region of PGR is not associated with endometriosis. However, further research is warranted to clarify the role of the +331G/A variant in endometriosis.

### The expression of PR-A and PR-B isoforms via +331G/A polymorphism in endometriosis

We observed a significant association between the +331G/A polymorphism and the expression level of PR isoform. Our results showed that patients with GA genotype had significantly high expression level of PR-B compared to patients with GG genotypes in +331G/A promoter polymorphism. The effects of progesterone depend on tight regulation of the PR-A and -B isoform balance. Altered PR-A/PR-B ratio modifies progesterone action via differential regulation of specific progesterone response targeted genes that may progress endometriosis disease. Progesterone reduces the expression of pro-inflammatory genes when the PR-A/PR-B ratio favored PR-B and improves these gene expressions by favoring PR-A isoform (21, 30). As a consequence, the specific genomic effect of progesterone responsiveness is equally related to the PR-A/PR-B ratio and PR-A isoform is an endogenous repressor of PR-B (8). Attia et al have reported a decreased concentration of PR-B isoform in ectopic lesions, which would be consistent with a protective role of +331A allele that increase PR-B isoform level and therefore may could compensate progesterone responsiveness (9, 11). Consequently, the presence of the +331A allele is hypothesized to lead to a greater effect of progesterone. The increased production of PR-B and the decreased level of PR-A isoforms by the +331A allele may be able to influence the function of progesterone via PR-A/PR-B ratio alternation and reduce the liability and symptoms of endometriosis.

## 5. Conclusion

In summary, we were interested to evaluate the correlation between the differential expression of PR isoforms via +331G/A promoter polymorphism of *PGR* in endometriosis. Although we did not find any association between the +331G/A SNP and endometriosis, we observed a significant relation between expression level of PR isoform and +331G/A polymorphism in endometriosis. It is known that endometriosis affects the function of oocyte and sperm through hampering the crosstalk between oocytes and sperms during fertilization by decreasing sperm binding to the zona pellucida. This hypothesis may be correlated further with inflammation activities due to progesterone resistance in endometriosis. We found overexpression level of PR-B via +331G/A polymorphism, as a consequence of altered PR-A/PR-B ratio in endometriosis. This alternation may be able to affect the function of the follicular microenvironment, oocyte, and embryo cross-talking quality that may be able to reduce infertility in endometriosis.

##  Conflict of Interest

The authors have no conflict of interests.
